# The Effects of Graded Levels of Calorie Restriction: XVIII.Tissue-Specific Changes in Cell Size and Number in Response to Calorie Restriction

**DOI:** 10.1093/gerona/glac110

**Published:** 2022-05-26

**Authors:** Daniel Phillips, Hayleigh Mathers, Sharon E Mitchell, John R Speakman

**Affiliations:** School of Biological Sciences, University of Aberdeen, Aberdeen, UK; School of Biological Sciences, University of Aberdeen, Aberdeen, UK; School of Biological Sciences, University of Aberdeen, Aberdeen, UK; School of Biological Sciences, University of Aberdeen, Aberdeen, UK; Shenzhen Key Laboratory of Metabolic Health, Center for Energy Metabolism and Reproduction, Shenzhen Institutes of Advanced Technology, Chinese Academy of Sciences, Shenzhen, China

**Keywords:** Calorie restriction, Hyper/hypoplasia, Hyper/hypotrophy, Tissue utilization, Weight loss

## Abstract

Calorie restriction (CR) without malnutrition increases the health and life span of diverse taxa. The mechanism(s) behind CR are debated but may be directly linked to body composition changes that maintain energy balance. During a deficit, energy is primarily obtained from white adipose tissue (WAT; utilized) while other tissues remain unchanged (protected) or grow (invested) relative to body mass. The changes in mass of 6 tissues from 48 male C57BL/6 mice following 3-month graded (10%, 20%, 30%, or 40%) CR or fed ad libitum for 12 or 24 hours a day were related to cell size (hypo/hypertrophy) and/or number (hypo/hyperplasia). Tissues studied were retroperitoneal and subcutaneous WAT, brown adipose tissue (BAT; utilized), lungs (protected), and stomach and cecum (invested). Methodology was based on number of nuclei/tissue equaling the number of cells. Extracted DNA was quantified and used to estimate cell numbers (total DNA/DNA per diploid nucleus) and size (tissue mass/nuclei number). WAT utilization was caused solely by hypotrophy whereas BAT utilization resulted from reduced cell number and size. WAT cell size positively correlated with circulating hormones related to energy balance, and BAT cell number and size positively correlated with body temperature. No changes were found in the lungs, consistent with their protected status, whereas hyperplasia appeared to be the dominant mechanism for invested alimentary-tract tissues. These findings indicate the pattern of change of cell size and number across increasing levels of short-term CR is tissue-specific.

Calorie restriction (CR) involves reduced intake of calories while avoiding malnutrition and has emerged as the most effective, reliable, and widely applicable nongenetic intervention for improving animal life and health span (1). Although several questions are still debated (2), one outcome has been consistently observed in most rodent models: there is a positive, linear correlation between the degree of CR and both average and maximal life span (1). To date the maximum CR level tested was 65% (3), beyond which there is presumably a rapid decline in health and life span due to utilization of functionally essential tissues. Benefits are greatest when initiated shortly after weaning, although exact values differ with strain, protocol, and genotype, with CR even reducing health and/or life span on some genetic backgrounds (1,4). CR is also linked to several whole animal- and molecular-level changes in physiology and behavior. These include increased hunger (5), cognitive performance (6), and autophagy (7), as well as reduced body temperature (8), blood glucose, insulin and insulin-like growth factor (IGF-1) levels (9), and oxidative stress (10). CR is also emerging as being protective against detrimental senescence and inflammatory senescence-associated secretory phenotype of cell populations implicated in aging (11).

Of particular historical interest has been the reduction of resting metabolic rate (RMR) caused by CR at the level of the whole organism, which contributes to accommodating the lowered food supply to maintain energy balance. Indeed, one theory regarding CR-extended life span is that it is a direct result of suppressed metabolism (12). This is consistent with the fact that, because whole-body RMR inversely correlates with the level of CR, and greater CR extends life span, whole-body RMR strongly correlates with CR-related life-extension. However, as has been pointed out previously (13), there exists a serious problem surrounding the calculation and interpretation of animal RMR in the context of CR studies. This issue is that CR shifts the balance between ingested and expended calories. To bridge the gap, energy is primarily withdrawn from the body’s main energy reserve: white adipose tissue (WAT). While WAT has a greater energy density than lean body tissue, all tissues contain metabolizable energy in the form of fat and protein and can be differentially utilized to alleviate the energy deficit. Accordingly, as well as RMR, CR also leads to reduced body mass (BM) and changes in body composition which will confound accurate interpretations of RMR if not taken into consideration. Appropriate estimates of RMR under CR, therefore, require detailed morphological information about the animals being studied which can be used to accurately correct for BM and composition, and thus determine if reduced RMR reflects metabolic suppression, or simply the accompanying changes in morphology (but see (14,15)). The extent to which different tissues are utilized can be calculated from the gradient of the relationships between Log organ mass and Log total BM (16). A value >1 on this index reflects disproportionate utilization while a value of 1 suggests isometric use, values between 0 and 1 suggest that tissue is under-utilized or “protected,” and values <0 suggest energy is being “invested” into the tissue. Mitchell et al. (16) previously used this method to provide a detailed quantitative analysis of differential tissue utilization in male C57BL/6 mice under short-term (3-month) graded (10%, 20%, 30%, and 40%) CR. They reported that WAT stores have utilization values >1, suggesting preferential utilization, tissues from the digestive tract have values <0, indicating energy is being invested in them, whereas vital organs had values > 0 < 1, and hence were protected.

The utility of this detailed survey of changing body composition under short-term graded CR (16) has proven to be twofold. First, it strengthens the existing schema of tissue utilization under CR, which is that an initial discrepancy between calorie intake and expenditure is offset by mobilizing energy stores in (primarily visceral) WAT fat depots (17). Thereafter, lean BM and some tissues are increasingly utilized in concurrence with short-term growth (investment) of the digestive tract to increase nutrient absorption (18). Second, Mitchell et al. (13) used models informed by their detailed compositional data (16) to show that existing reports of suppressed RMR during CR (19,20) potentially resulted from inadequate, low-resolution models used to correct for the morphological changes associated with CR. Namely, when tissue mass changes are accurately accounted for, the reduced RMR of mice under CR could be fully explained without the need to infer additional physiological changes (13). However, in addition to contributing to the debate surrounding the question of how CR affects murine RMR, these compositional changes raise the independent question of which mechanism(s) underpin the dynamic changes in tissue mass during short-term CR.

Regulated changes in tissue mass generally occur as a result of 2 main mechanisms; increased (hyperplasia) or decreased (hypoplasia) cell numbers, or increased (hypertrophy) or decreased (hypotrophy) cell size (21). The aim of the present study was to determine the extent to which changes in the mass of 6 tissues under CR were caused by alterations in either cell size and/or number. Of the tissues studied, 3 were utilized (retroperitoneal and subcutaneous white adipose tissue [rWAT and scWAT] and brown adipose tissue [BAT] depots), 1 protected (lungs), and 2 invested (stomach and cecum) during 3-month graded (10%, 20%, 30%, and 40%) CR in C57BL/6 male mice (16). Cell size and number were then correlated with factors indicated to potentially play a role in health/life span, such as various hormones and body temperature.

## Method

### Experimental Design and Animal Work

Animal procedures were reviewed and approved by the University of Aberdeen ethics approval committee and conducted under a Home Office issued license compliant with the Animals (Scientific Procedures) Act 1986. All procedures and methodology used were previously reported (16). Briefly, 48 20-week-old male C57BL/6 mice were randomly assigned to one of four (10%, 20%, 30%, or 40%) experimental CR groups or one of two (24- or 12-hour ad libitum fed; 24AL and 12AL, respectively) control groups, and matched for BM; 24AL (*n* = 9); 12AL (*n* = 8); 10CR (*n* = 8); 20CR (*n* = 8); 30CR (*n* = 7); and 40CR (*n* = 9). CR was initiated when mice were 20 weeks-old and developmentally mature thus limiting the impact of CR on developmental processes. All CR and 12AL animals were fed just once a day immediately before lights out (18:30 hour). Food was removed at 06:30 from 12AL.

After 3-month of CR (at ~8 months old), all animals were killed, and their tissues were promptly dissected, weighed accurately to 0.00001 g (Sartorius R200D), then flash-frozen in liquid nitrogen and stored at −80°C. Six tissues were chosen for analysis, each already characterized as being either utilized, protected, or invested under CR (16). The relative utilization of the tissues was previously reported (16) and calculated by plotting the Log_e_ of dissected organ weight against the Log_e_ of final BM across all individuals. A linear least squares fit regression was fitted and the gradient (β) used. An organ with a gradient β = 1 would be utilized at the same rate as total BM under CR. An organ with β > 1 declined more rapidly than BM therefore was preferentially utilized under CR. In contrast, organs where β > 0 < 1 were relatively protected. Any organ where β < 0 increased in weight as the animals decreased in body weight, that is, energy was being invested in them. The utilized tissues chosen were the rWAT, scWAT, and BAT. The protected tissue was the lungs, and the invested tissues were the stomach and cecum. Cell size and number were based on the assumption of a single nucleus per cell (22) and would therefore be compromised by polyploidy. However, to the best of our knowledge the selected tissues do not display polyploidy in adult mice (23).

### DNA Extraction

DNA was extracted from tissue samples using the NucleoSpin Tissue (Macherey-Nagel, Germany) kit following suppliers’ instructions. To account for heterogeneity of cells within the tissues 2 random samples were taken from each tissue (12.5~30 mg; Sartorius R200D) and analyzed in duplicate. All samples were reconstituted in 60 μL water and then quantified and qualified using NanoDrop spectrophotometer (Thermo Scientific, Waltham, MA, UK).

### Determination of Cell Number and Size

Calculations of cell number and size for each tissue sample were carried out using equations adapted from (22). Because nuclear DNA content remains constant in most organs (about 6.03 pg/diploid nucleus), and most eukaryotic nonmitotic cells are limited to a single nucleus in mice (except for striated muscle cells), the number of nuclei per tissue was considered equal to the number of cells. Concentrations of DNA (mg) per 60 μL were used to calculate DNA (mg) per tissue sample, $\frac{mg.\;DNA\;per\;60\;\mu \;L}{Weight\;of\;Sample\;\left(mg\right)},$ and subsequently the total DNA (mg) of each tissue, (mg) DNA per mg. of Sample Weight ×Weight of Tissue (mg). Given the calculated total mg DNA of each tissue, the number of nuclei within each tissue (expressed in millions) was calculated using $\frac{(mg.\;DNA\;per\;Tissue\times 10^{3})}{6.03},$ where 6.03 is the (pg) DNA content of individual male C57BL/6 mice nuclei (24). Outlying duplicates (>30% coefficient of variance [CV]) were identified and (25/546) removed. An average for the duplicates was then obtained for each animal. Due to some missing samples or problems with extraction final tissue numbers were rWAT: *n* = 45, scWAT: *n* = 47, and BAT: *n* = 45, lungs *n* = 46, stomach *n* = 42, and cecum *n* = 48. Average cell weight (weight per nucleus; ng) was calculated using the total mass and number of nuclei of each tissue, $\frac{Weight\;of\;Organ\left(g\right)\times 10^{3}}{Nuclei\;per\;Organ\;(in\;millions)},$ and used as a proxy of cell size.

### Statistical Analysis

Multiple least square linear regressions were first used to associate nuclei (ie, cell) numbers and weight (ie, cell size) to their respective wet tissue masses (α = 0.05). Then, to determine the response of tissues to short-term graded CR, cell number and size data for each tissue were regressed against the level of CR taken as a continuous variable (0%–40% CR, where 12AL represented 0%) using either univariate or multiple linear regressions (α = 0.05). Further analysis was conducted to additionally relate tissue cell size and numbers to body temperature and previously measured levels of circulating proinflammatory cytokines (tumor necrosis factor 1α; TNFα, interleukin 6; IL6) and hormones regulating tissue (insulin growth factor-1; IGF-1) and adipose (insulin) expansion, insulin resistance (resistin), and energy homeostasis (leptin) in the same mice (25), using Pearson correlations or multiple linear models (*adj*.*p* < .05 after correcting for number of hormones). Results are presented as group mean ± standard deviation from the mean. All statistical tests were carried out in Minitab V.19 or RStudio 3.6.3 and visualized using the ggplot2 R package.

## Results

### Utilized Tissues

**Figure 1.**
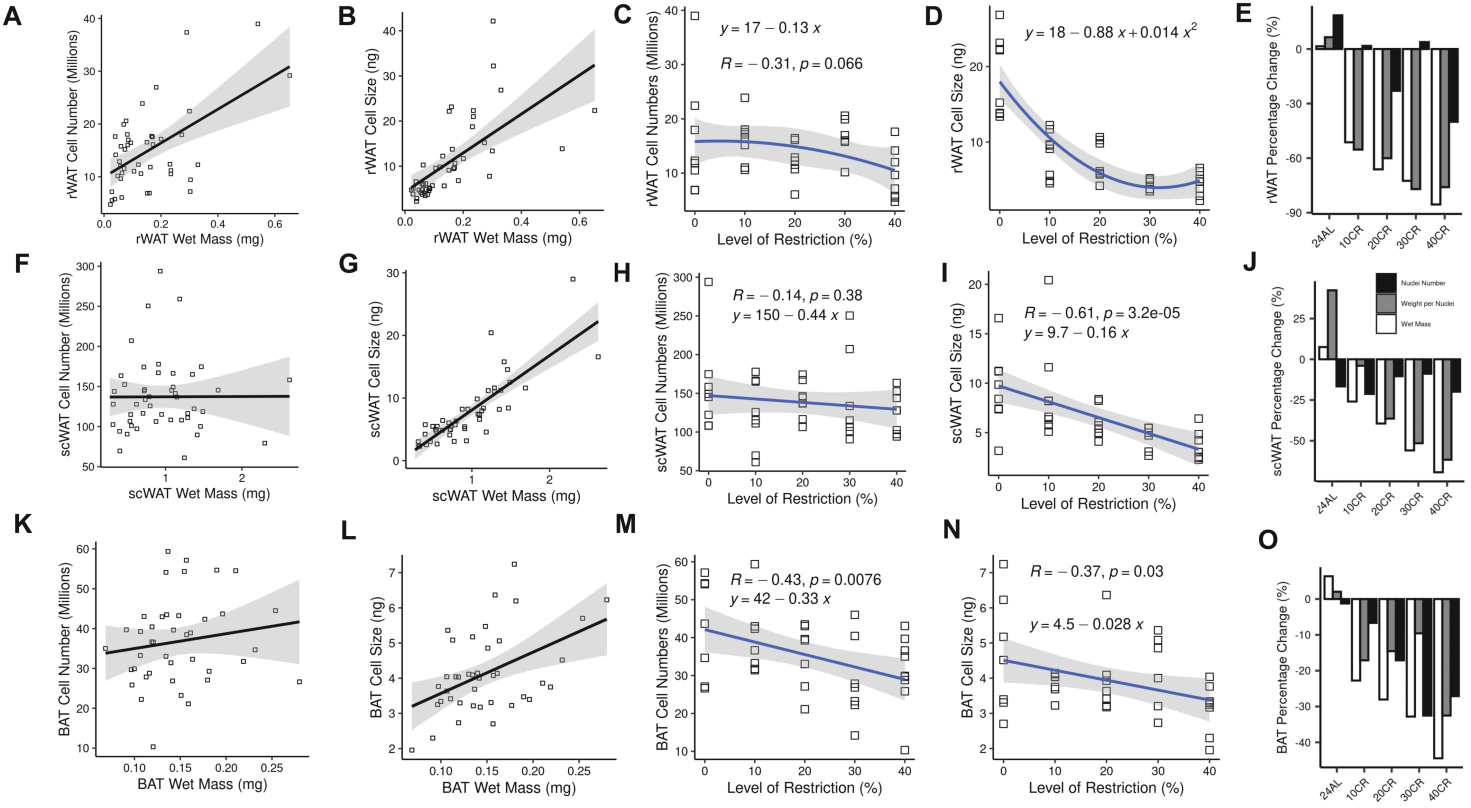
Tissue utilization in 3 adipose tissues which were differentially utilized in response to 3 months graded (10%–40%) calorie restriction (CR) (16). Associations between both nuclei per organ (millions: cell numbers) and weight per nucleus (ng: cell size) and wet tissue mass, the relationship between cell number and size with level of restriction, and percentage change relative to the 12-hour ad libitum (12AL) control group for the retroperitoneal white adipose tissue (rWAT; **A, B, C, D, E**), subcutaneous white adipose tissue (scWAT; **F, G, H, I, J**), and brown adipose tissue (BAT; **K, L, M, N, O**) depots of mice at increasing levels of CR, respectively (16).

While both cell number (*t* = 11.31, *p* < .001; Figure 1A) and size (*t* = 12.73, *p* < .001; Figure 1B) significantly predicted increased rWAT wet tissue mass (multiple regression: *F*_2,42_ = 126.6, *p* < .001, *adj.r*2 = 0.85), there was no significant effect of CR level on cell numbers in the rWAT (least squares regression: *R* = −0.031, *p* = .066; Figure 1C), but a significant reduction in cell size (*F*_2,34_ = 44.96, *p* < .001, *adj.r*^2^ = 0.73) best explained by a second-degree polynomial equation (*y* = 18 − 0.88*x* + 0.014*x*^2^; Figure 1D). This pattern of rWAT hypotrophy, therefore, followed the reduction in rWAT wet mass (Figure 1E).

Similarly, in the scWAT both cell number (*t* = 6.67, *p* < .001; Figure 1F) and especially size (*t* = 15.29, *p* < .001; Figure 1G) significantly predicted wet tissue mass (multiple regression: *F*_2,44_ = 116.9, *p* < .001, *adj.r*^2^ = 0.83). CR did not have any detectable effect on cell numbers in the scWAT (least squares regression: *R* = −0.14, *p* = .38; Figure 1H), but resulted in a linear (*y* = 9.7 − 0.16*x*) reduction in scWAT cell sizes (*F*_1,37_ = 22.43, *p* < .001, *adj.r*^2^ = 0.36; Figure 1I). This reduction in scWAT adipocyte size appropriately matched the decrease in scWAT wet mass (Figure 1J).

BAT cell numbers (*t* = 2.6, *p* = .013; Figure 1K) were weakly associated with wet tissue mass whereas BAT cell sizes (*t* = 4.22, *p* < .001; Figure 1L) showed a significant and strong positive association. Both BAT cell number (*F*_1,35_ = 8.04, *p* = .008, *adj.r*^2^ = 0.16; Figure 1M) and size (*F*_1,33_ = 5.159, *p* = .03, *adj.r*^2^ = 0.11; Figure 1N) were significantly reduced with increasing CR. These reduced BAT cell sizes and numbers are in line with reductions in BAT wet mass in the same mice (Figure 1O).

**Figure 2.**
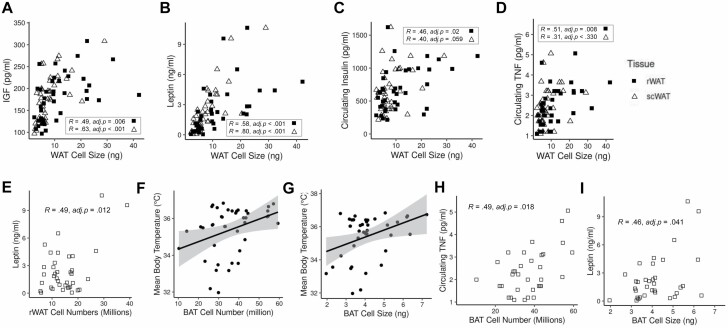
Scatter plots showing significant associations between retroperitoneal and subcutaneous white adipose tissue (rWAT and scWAT, respectively) cell sizes and circulating (**A**) insulin-like growth factor-1 (IGF-1), (**B**) leptin, (**C**) insulin, (**D**) tumor necrosis factor α (TNF-α), and (**E**) rWAT cell numbers and circulating leptin. (**F** and **G**) show associations between mean body temperature and brown adipose tissue (BAT) cell numbers and size, respectively, and (**H** and **I**) show associations between BAT cell numbers and size and circulating TNF-α and leptin, respectively. Circulating hormones (25) and mean body temperature (8) data originates from previously published work on the same mice responding to 3-month 10%–40% calorie restriction (16).

To explore the physiological relevance of WAT utilization, WAT cell size and number data for each mouse were correlated with metabolic- and life span-related markers (8,25) (See Method: Statistical Analysis). The cell size in both rWAT (Pearson’s correlation: *R* = 0.049, *adj.p* = .006; *R* = 0.58, *adj.p* < .001) and scWAT (Pearson’s correlation: *R* = 0.63, *adj.p* < .001; *R* = 0.8, *adj.p* < .001) correlated positively with circulating IGF-1 and leptin, respectively (Figure 2A and B), with rWAT cell sizes alone exhibiting an additional positive correlation with circulating insulin (*R* = 0.46, *adj.p* = .02) and TNF-α (*R* = 0.51, *adj.p* = .008; Figure 2C and D). The only significant association with WAT cell numbers was in the rWAT, where cell numbers correlated with circulating leptin (*R* = 0.49, *adj.p* = .012; Figure 2E).

The utilization of the BAT was further studied in relation to previous data showing reduced body temperature with increasing CR in the same mice (8). Using multiple regression both BAT cell number (*t* = 4.53, *p* < .001; Figure 2F) and size (*t* = 4.62, *p* < .001; Figure 2G) predicted the mean body temperature of mice responding to CR (multiple regression: *F*_2,37_ = 14.7, *p* < .001, *adj.r*^2^ = 0.41). BAT cell numbers (*R* = 0.49, *adj.p* = .018; Figure 2H) and size (*R* = .46, *adj.p* = .041; Figure 2I) were additionally correlated with increased circulating TNF-α and leptin, respectively.

### Protected Tissue

**Figure 3.**
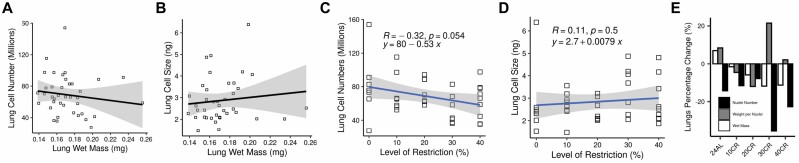
Changes in wet mass of lung in response to calorie restriction (CR) was not associated with (**A**) nuclei per organ (millions: cell numbers) and (**B**) weight per nucleus (ng: cell size). There was no relationship between wet tissue mass, cell number (**C**) and size (**D**) and level of calorie restriction (CR). (**E**) percentage change relative to the 12-hour ad libitum (12AL) control group (16) in mice responding to 3-month graded (10%–40%) CR.

In the lungs, neither cell number nor size were related to wet tissue mass (multiple regression: *F*_2,36_ = 0.38, *p* = .688; Figure 3A and B) or the level of CR (cell number linear regression: *R* = −0.32, *p* = .054; cell size linear regression: *R* = −0.11, *p* = .5; Figure 3C and D), consistent with stable mass of this tissue during CR (Figure 3E).

### Invested Tissues

**Figure 4.**
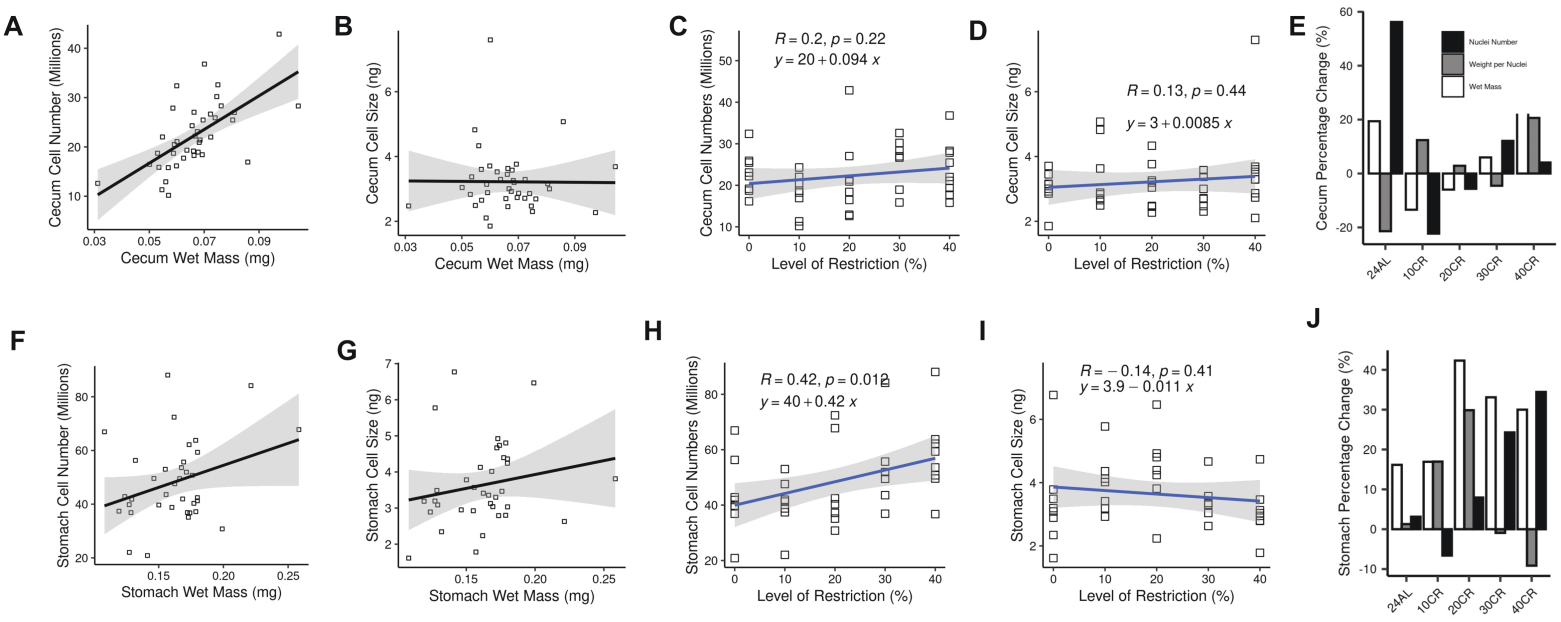
Tissue utilization in 2 invested tissues (cecum and stomach) in response to graded (10%–40%) calorie restriction. Associations between both nuclei per organ (millions: cell numbers) and weight per nucleus (ng: cell size) and wet tissue mass, cell number and size and level of restriction, and percentage change relative to the 12-hour ad libitum (AL) control group for the cecum (cecum; **A, B, C, D, E**) and stomach (stomach; **F, G, H, I, J**) of mice responding to 3-month graded (10%–40%) calorie restriction (16).

In the cecum, cell number (*t* = 5.82, *p* < .001; Figure 4A) showed a strong positive association with wet tissue mass, with cell size exhibiting a much weaker association (*t* = 2.40, *p* = .021; multiple regression: *F*_2,37_ = 16.92, *p* < .001, *adj.r*^2^ = 0.45; Figure 4B). Both cecal cell number (regression: *R* = 0.2, *p* = .22; Figure 4C) and cell size (regression: *R* = 0.13, *p* = .44; Figure 4C) were unaffected by CR level (16) (Figure 4E).

In the stomach, when considered together as part of a multiple regression model, both increased cell number (*t* = 7.15, *p* < .001; Figure 4F) and size (*t* = 6.76, *p* < .001; Figure 4G) were significantly associated with stomach wet mass (*F*_2,33_ = 27.19, *p* < .001, *adj.r*^2^ = 0.6). Moreover, graded CR resulted in a significant increase in cell numbers (regression: *F*_1,34_ = 7.1, *p* = .012, *adj.r*^2^ = 0.15; Figure 4H) but had no effect on cell size (regression: *R* = −0.14, *p* = .41; Figure 4I and J).

**Figure 5.**
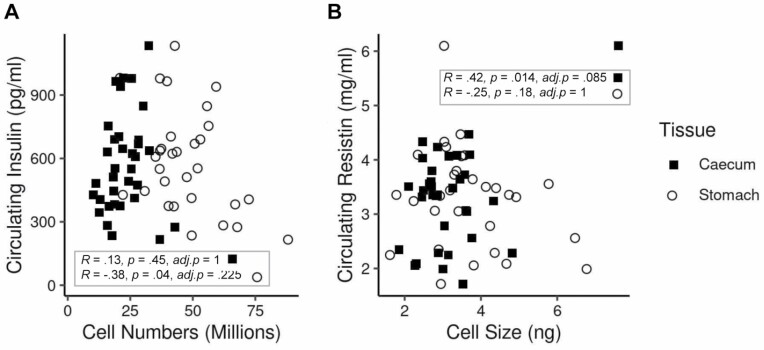
Scatter plots showing associations between cell number and size in both the cecum and stomach and (**A**) circulating insulin and (**B**) circulating resistin in mice responding to 3-month graded (10%–40%) calorie restriction (CR) (16).

In addition to their response to CR, cecum and stomach cell size and numbers were tested for association with several metabolic hormones. While stomach cell number (Pearson’s correlation: *R* = −0.38, *p* = .037, *adj.p* = .225) and cecum cell size (Pearson’s correlation: *R* = 0.42, *p* = .01, *adj.p* = .085) had a nominally significant negative correlation with circulating insulin and positive correlation with circulating resistin, respectively, these were not robust to multiple testing corrections (Figure 5A and B).

## Discussion

The process of animal growth and somatic maintenance is fundamentally predicated on the continuous consumption and assimilation of food. Accordingly, Theodosius Dobzhansky (26) famously and aptly referred to the body as “a conglomeration of transformed groceries.” However, if an animal’s food intake is restricted it faces an energy deficit that must be accommodated if it is to continue to maintain itself and survive. How animals respond to this challenge in the context of CR studies has attracted particular attention due to the fact that such responses do not merely maintain the body but extend life. Although an energy deficit can be somewhat offset behaviourally by reducing physical activity to limit energy expenditure (27), the usual response to such an imposed energy deficit is to also utilize energy stored in the body in the form of both lean and fat tissue. Hence, the most universal outcome of CR studies is a reduction in total BM. However, in remedying the energy deficits caused by CR, tissues are not utilized uniformly, but are instead differentially exploited as the level of CR increases, leading to significant changes in body composition (16). Here it is demonstrated that these changes in body composition are mediated by tissues responding to graded CR by independently altering the size and/or number of their cells.

Three tissues that were preferentially utilized relative to BM were investigated. These were the rWAT, scWAT, and BAT depots. Relative to BM, the wet mass of these tissues decreased linearly with increasing CR, with a max reduction compared to the 12AL control group of 86%, 69%, and 44% at 40CR for the rWAT, scWAT, and BAT, respectively (16). The rWAT and scWAT (along with epididymal WAT) depots were the most utilized tissue types overall, whereas BAT depots were less utilized. Although both WAT cell number and size were both associated with tissue mass (Figure 1A, B, F, and G), the present work indicates that the preferential utilization of rWAT and scWAT following CR was caused solely by a reduction in cell size (Figure 1D and I), but cell numbers were nearly significant (*p* = .066) in rWAT. These results are consistent with studies reporting reduced adipocyte sizes in scWAT depots of male C57BL/6 and BALB/c mice at 40CR (28) and rWAT depots of male Wistar rats at 30CR (29).

WAT is separated into subcutaneous and visceral depots, sometimes referred to as “good” and “bad” fat. Higher levels of visceral fat, rWAT, and epididymal depots are associated with insulin resistance, and surgical removal of visceral fat has been shown to extend life span (30). Conversely, scWAT, is associated with improved insulin-sensitivity, and, notably, long-lived mouse models have larger scWAT depots than shorter-lived strains, indicative of its protective effect on metabolic health (31). The hypotrophic effect of CR was greatest in the rWAT, where there was a sharp negative polynomial decline (Figure 1D) with a max ~77% reduction in cell size at 30CR relative to 12AL controls (Figure 1E), compared to a linear reduction (Figure 1I) and lesser maximal reduction of ~62% in the scWAT at 40CR (Figure 1J). Accordingly, differential utilization of these different WAT depots may be a contributing mechanism to the improved glucose homeostasis observed in these same mice following graded CR (25). This effect is consistent with the proposed existence of an allometric scaling law dictating that visceral WAT will be consistently utilized at a greater rate than total fat mass regardless of the implemented weight loss strategy (32).

WAT hypotrophy is often suggested as a parsimonious explanation of many health benefits of CR. Hypotrophic white adipocytes secrete more adiponectin and less inflammatory cytokines (eg, leptin) than their hypertrophic counterparts (33) (Figure 2B–D), as well as exhibiting increased insulin-sensitivity (Figure 2C) and lipid-buffering activities (34). Similarly, scWAT is known to secrete more leptin than rWAT (35), a fact further supported in the present work (Figure 2B). Although, it should be noted that leptin was also associated with rWAT, but not scWAT, cell numbers (Figure 2E). scWAT also acts to generally improve glucose metabolism (36) whereas excess rWAT is linked to metabolic syndrome and aging (37). Indeed, Liao et al. (38) found that CR was more likely to extend life span in mice strains that protected their fat stores and suggested that some of this benefit may have arisen from the redistribution of subcutaneous fat stores over visceral ones, as has been found in C57BL/6 females (39). Previous studies have tended to investigate the role of changes in WAT cell size without reference to concurrent reductions in absolute adipocyte numbers (28,29), whereas here strong evidence for rWAT and scWAT hypotrophy is complemented by evidence for a lack of hypoplasia. Although one experiment reported a reduction in rWAT cell numbers in Fischer rats following lifelong 40CR compared to controls (40), it is important to highlight that this study concerned the differential growth of rWAT depots across the whole lifetime, not an induced reduction in cell numbers in direct response to short-term CR. Reduced hyperplasia is not the same as CR-induced hypoplasia, nor does it necessarily share similar physiological mechanisms or implications for CR-extended life.

BAT mass of these mice was protected from utilization at 10CR but significantly reduced relative to the 12AL group at 20CR (−28%), 30CR (−33%), and 40CR (−44%) (16). Unlike in the WAT, BAT utilization was associated with a combination of hypotrophy and hypoplasia (Figure 1M and N). However, given that BAT cell numbers were only weakly correlated with wet tissue mass (Figure 1K), hypoplasia is likely to be the main cause of utilization. Because BAT cell numbers and size are positively associated with body temperature (Figure 2F and G), reduced BAT cell size and numbers seems at odds with the higher incidences of torpor reported in these same mice at 40CR (8). However, the increased readiness to enter torpor may in part be related to the reduction in BAT cell sizes, as this would be predicted to result in an associated reduction in circulating leptin (Figure 2I), which has previously been identified as an important signal initiating torpor in these mice (8). It should also be noted that the reduced BAT cell numbers would also be predicted to be associated with in a decrease in TNFα (Figure 2H), which has an inhibitory effect on thermogenesis (41), and which may thus partially alleviate any concurrent reduction in thermogenic capacity caused by BAT utilization. Furthermore, these results indicating both BAT hypotrophy and hypoplasia are seemingly inconsistent with previous CR studies noting an increase in BAT mass or proliferation in both mice and rats (15,42). Although, more recently a study reported that 40% CR reduced BAT mass in C57BL/6J male mice, but only in young mice not old or middle-aged ones, and also did not find any change in BAT morphology (43). Given the central role of BAT in thermogenesis and energy homeostasis, clarifying the reasons behind these inconsistent results and elucidating the factors driving the responses of BAT to CR will be a key future goal.

This study investigated one tissue, the lungs, which was protected from significant utilization relative to total BM following CR (16). Consistent with this picture, no significant response to graded CR was shown by either lung cell numbers or size (Figure 3A–E). Elucidating the impact of CR on mammalian lung structure and function is important because, in addition to bone marrow, the lungs are one of few organs that may be negatively affected by CR (1). For example, Massaro et al. (44) reported a reduction in alveoli number and surface-area (larger but thinner alveoli) in C57BL/6 mice in response to just 15 days of CR, and in a review of related data (45)suggested CR may therefore increase the risk of “nutritional emphysema.” Indeed, 35% of alveoli were lost within the first 72 hours of CR, with a further 12% reduction in the following 12 days, although there was no reported reduction in total lung- or gas-exchange air-volume (44). Conversely, in a similar C57BL/6 model of CR (see later), Bishai and Mitzner (46) reported no change in alveoli size (or cord length) but a significant reduction in lung capacity and also evidence of reduced alveoli numbers compared to AL control mice.

It is currently unclear why changes in cell size and number were not recorded in the lungs in the present study, whereas elsewhere, such changes have been reported to occur in mice of the same strain undergoing CR. Moreover, while the lungs of these mice were relatively protected from utilization considered alongside a reduction in total mass, considered alone there was still a significant reduction of 11% at 40CR relative to 12AL (16). One possible explanation relates to differences in the level(s) of CR studied. Mitchell et al (16) reduced the intake by 10%, 20%, 30%, or 40% of their previous AL intake, whereas both Massaro et al. (44) and Bishai and Mitzner (46) restricted the intake of their CR mice by one third (ie, ~66CR, in the terminology of this article) of their AL intake. Moreover, the aforementioned studies used morphometric analyses of tissue micrographs to assess morphology, whereas here, changes were evaluated using lung sample DNA content. Indeed, Bishai and Mitzner (46) discuss the potential limitations of morphometric analyses of lung histology, referencing both the many different ways of counting cell (ie, alveoli) numbers and the inherent variability of lung tissue physiology as potential sources of bias. While this limitation is much less likely to apply here due to changes in cell size and number being inferred from the DNA content of relatively large tissue samples, we cannot evaluate functionality. It is also important to note that even where reductions in lung mass or alveoli number are reported, this may be given the reduced oxygen requirement under CR. Accordingly, the impact of CR on the lungs in terms of functionality remains an open question.

Two tissues that were invested in relative to BM following CR were investigated in the present study. These were the cecum and the stomach. There has been considerable research on the comparative function and plasticity of the cecum in the context of animal nutrition due to the important role it plays in the absorption of fluids. However, surprisingly, very little work directly studies ceca cells in the context of CR. In the mice studied here, the cecum, along with all other tissues of the digestive tract, was shown to increase in mass in response to CR, with a maximum mass increase of ~25% in 40CR mice compared to the 12AL control group (16). Regression analyses did not indicate any response in cecal cell numbers or size to CR (Figure 4C and D). However, evidence supported the main utilization effect likely being hyperplasia, because there was a strong positive correlation between ceca mass and cell numbers, but only a weak correlation regarding cell size (Figure 4A and B). Nonetheless, the functional consequences of cecal hyperplasia during CR remain unclear, as neither cell numbers, or size were significantly associated with any circulating hormones.

Understanding how the stomach responds to CR is important because there is evidence it provides a physiological mechanism to reduce the severity of restriction by improving nutrient absorption (18). The stomachs analyzed in the present study exhibited a maximum wet mass increase of 42% at 20CR compared to 12AL controls, which was a significant investment when corrected for change in BM (16). While both cell number and size were related to stomach mass (Figure 1F and G), regression analysis provided evidence that the changes in stomach mass during CR resulted solely from an increase in cell numbers (Figure 4F). These results suggesting cecal hyperplasia following CR appear inconsistent with work by Yang et al. (18), who reported an increased stomach mass of female C57BL/6 mice following lifelong 40CR which they determined to be caused largely by forestomach hypertrophy. However, they also noted an increased mass of the stomach antrum but could not relate this to hypertrophy, possibly because it was caused by hyperplasia, which was not studied.

In conclusion, this study determined that WAT responded to graded CR exclusively by reducing cell size, whereas BAT was utilized by reducing both cell size and numbers. Furthermore, although WAT hypotrophy was proportional to CR level, the rate of reduction was unique to each specific depot, with rWAT responding more sharply to the energy deficit than scWAT, consistent with fat redistribution as a factor in the health and life-span benefits of CR. This study also confirmed a lack of change in cell size or number in the lungs in line with their previously reported protected status in the same mice, potentially challenging the idea that CR is detrimental to mammalian lung function. Differential investment in stomach mass during graded CR was associated with an increase in stomach cell numbers. Conversely, investment in the cecum during graded CR could not be associated with either cell numbers or size. However, correlations of both cell size and number with cecal wet tissue mass implicated hyperplasia more strongly than hypertrophy in cecal investment following graded CR. Overall, this study has added greater resolution to the picture of how animals utilize tissues to restore energy balance during short-term CR and related the mechanisms mediating utilization to associated changes in physiology known to be important to metabolism and health span.

### Limitations

There are several limitations to the methodology used. First, it cannot be assumed that the response to all utilized, protected, or invested tissues would follow the patterns reported here. Indeed, we found significant differences in response within the three “utilized” tissues. We were also unable to evaluate cell turnover. Hence, relative contributions of proliferation and cell loss were not accounted for. Cell populations may have turned over, and proportion of cell types changed. Furthermore, cell type cannot be determined by this method, and an influx or efflux of cells of a specific cell type, for instance, macrophages in the adipose tissue, could result in very different interpretations. Senescent or quiescent cells are also very likely to be present in the tissues measured. In fact, using qRT-PCR, we found a number of classical transcriptomic markers of senescent cells were downregulated with CR in the colon of the same mice, potentially suggesting a reduction in the number of senescent cells in the CR mice (47).

In addition, the method we used assumes a single nucleus, and therefore was not robust to polyploidy. Polyploidy in mice is time, tissue, and cell-specific with high levels found in liver and heart, particularly early in life (48). Polyploidy in mammals more generally has also been reported in skeletal muscle, placenta, brain, ovary, and bone marrow. We could not find reports of polyploidy in any of the tissues we studied, suggesting our assumption of one nucleus per cell may not be a serious limitation. Moreover, polyploidy appears limited to the period of development (48). While polyploidy has not been reported in any of the tissues studied here it is of interest that polyploidy in rat liver was reduced in rodents undergoing CR (49).
